# Tetra­kis(μ-2-phen­oxy­propionato)-κ^3^ *O*,*O*′:*O*′;κ^3^ *O*:*O*,*O*′;κ^4^ *O*:*O*′-bis­[(1,10-phenanthroline-κ^2^ *N*,*N*′)(2-phen­oxy­propionato-κ^2^ *O*,*O*′)terbium(III)]

**DOI:** 10.1107/S1600536811032041

**Published:** 2011-08-11

**Authors:** Jin-Bei Shen, Jia-Lu Liu, Guo-Liang Zhao

**Affiliations:** a College of Chemistry and Life Sciences, Zhejiang Normal University, Jinhua 321004, Zhejiang, People’s Republic of China; b Zhejiang Normal University Xingzhi College, Jinhua, Zhejiang 321004, People’s Republic of China

## Abstract

In the centrosymmetric binuclear title complex, [Tb_2_(C_9_H_9_O_3_)_6_(C_12_H_8_N_2_)_2_], the two Tb^III^ ions are linked by four 2-phen­oxy­propionate (*L*) groups through their bi- and tridentate bridging modes. Each Tb^III^ ion is nine-coordinated by one 1,10-phenanthroline mol­ecule, one bidentate carboxyl­ate group and four bridging carboxyl­ate groups in a distorted TbN_2_O_7_ monocapped square-anti­prismatic geometry.

## Related literature

For background to phen­oxy­alkanoic acids, see: Markus & Buser (1997 ▶). For a related structure, see: Zhao *et al.* (2008 ▶).
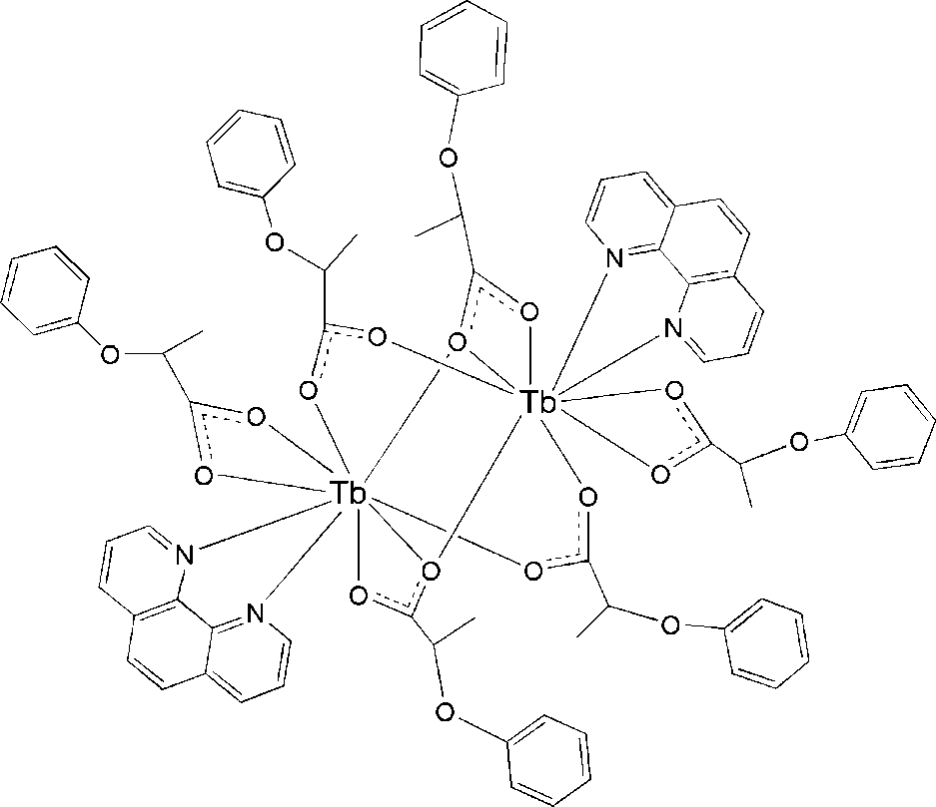


## Experimental

### 

#### Crystal data

[Tb_2_(C_9_H_9_O_3_)_6_(C_12_H_8_N_2_)_2_]
*M* *_r_* = 1669.24
Monoclinic, 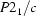

*a* = 11.4747 (3) Å
*b* = 25.8130 (8) Å
*c* = 13.8530 (3) Å
β = 120.585 (2)°
*V* = 3532.35 (16) Å^3^
*Z* = 2
Mo *K*α radiation
μ = 2.06 mm^−1^
*T* = 296 K
0.21 × 0.16 × 0.09 mm

#### Data collection

Bruker APEXII CCD diffractometer
Absorption correction: multi-scan (*SADABS*; Sheldrick, 1996 ▶) *T*_min_ = 0.685, *T*_max_ = 0.834
24708 measured reflections
6213 independent reflections
4431 reflections with *I* > 2σ(*I*)
*R*_int_ = 0.051

#### Refinement

*R*[*F* ^2^ > 2σ(*F* ^2^)] = 0.033
*wR*(*F* ^2^) = 0.073
*S* = 1.01
6213 reflections
464 parameters
H-atom parameters constrained
Δρ_max_ = 0.72 e Å^−3^
Δρ_min_ = −0.81 e Å^−3^

### 

Data collection: *APEX2* (Bruker, 2006 ▶); cell refinement: *SAINT* (Bruker, 2006 ▶); data reduction: *SAINT*; program(s) used to solve structure: *SHELXS97* (Sheldrick, 2008 ▶); program(s) used to refine structure: *SHELXL97* (Sheldrick, 2008 ▶); molecular graphics: *SHELXTL* (Sheldrick, 2008 ▶); software used to prepare material for publication: *SHELXL97*.

## Supplementary Material

Crystal structure: contains datablock(s) I, global. DOI: 10.1107/S1600536811032041/hb6349sup1.cif

Structure factors: contains datablock(s) I. DOI: 10.1107/S1600536811032041/hb6349Isup2.hkl

Additional supplementary materials:  crystallographic information; 3D view; checkCIF report

## Figures and Tables

**Table 1:** Selected bond lengths (Å)

Tb1—O8^i^	2.336 (3)
Tb1—O5^i^	2.344 (3)
Tb1—O4	2.381 (3)
Tb1—O2	2.422 (3)
Tb1—O7	2.457 (3)
Tb1—O1	2.475 (3)
Tb1—N2	2.525 (3)
Tb1—N1	2.606 (3)
Tb1—O8	2.623 (2)

Symmetry code: (i) 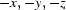.

**Fig. 1.**
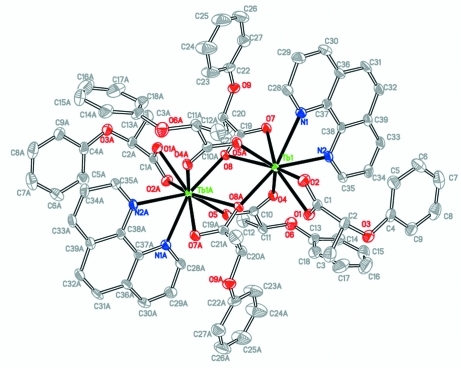
The molecular structure of (I) with displacement ellipsoids drawn at the 30% probability level. Atoms with suffix A are generated by (–x, –y, –z) and H atoms are omitted for clarity.

**Fig. 2.**
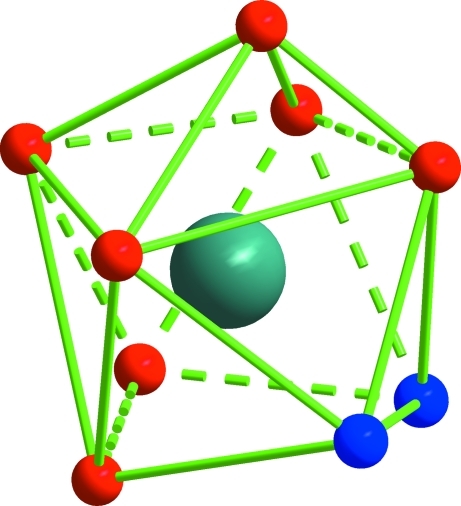
The coordination environment of the TbIII ion.

## supplementary crystallographic information

### Comment

The group of phenoxyalkanoic acids includes a considerable number of important herbicides. The desired biological activity is largely dependent on the length of the carbon chain of the alkanoic acid, the nature of the phenoxy group, and the position of its attachment to the carbon chain (Markus *et al.*, 1997). The fascinating structures of 2-phenoxypropionic acid complexes coupled with their special functionality catch our interests.Here,we describe the new title Tb^III^ complex, (I).

The structure of (I) is shown in Fig.1 and the coordination envi- ronment of Tb^III^ was shown in Fig. 2. The dimeric title compound(1) is centrosymmetric and is comprised of six *L* and two phen. The two Tb^III^ ions are linked together by four *L* groups through their bi- and tri- dentate bridging modes, form a dimeric unit with crystallographic inversion center, and the distance between two Tb^III^ ions is 3.9935 (3) Å, which is similar to the analogous complexe (Zhao *et al.*, 2008). Each Tb^III^ ion is coordinated to nine atoms, of which five oxygen atoms are from the bridging carboxylates, two oxygen atoms from the bidentate chelating carboxylate group, and two nitrogen atoms from a 1,10-phenanthroline molecule. The analysis of structural features indicates that the central Tb^III^ ion adopts a distorted mono-capped square antiprism geometry(Fig. 2). The Tb—O distances are all within the range 2.336 (3)–2.623 (2) Å, and the Tb—N distances rang from 2.525 (3)–2.606 (3) Å. The selected bond lengths for complex 1 are listed in Table 1.

### Experimental

Reagents and solvents used were of commercially available quality and without purified before using. 2-phenoxypropionic acid (1.5 mmol), Tb(NO_3_)_3_.6H_2_O (0.5 mmol) and 1,10-phenanthroline (0.5 mmol) were dissolved in 20 ml e nthanol, then 10 ml water was added to the above solution. The mixed solution was stirred for 12 h at room temperature. At last, deposit was filtered out and the colourless solution was kept in the open air to yield colourless blocks of (I) after several days.

### Refinement

The H atoms bonded to C and N atoms were positioned geometrically and refined using a riding model [aliphatic C—H =0.96 Å (*U*_iso_(H) = 1.5*U*_eq_(C)), aromatic C—H = 0.93 Å (*U*_iso_(H) = 1.2*U*_eq_(C))].

### Crystal data

**Table:**

[Tb_2_(C_9_H_9_O_3_)_6_(C_12_H_8_N_2_)_2_]	*F*(000) = 1680
*M**_r_* = 1669.24	*D*_x_ = 1.569 Mg m^−^^3^
Monoclinic, *P*2_1_/*c*	Mo *K*α radiation, λ = 0.71073 Å
Hall symbol: -P 2ybc	Cell parameters from 4163 reflections
*a* = 11.4747 (3) Å	θ = 1.9–25.0°
*b* = 25.8130 (8) Å	µ = 2.06 mm^−^^1^
*c* = 13.8530 (3) Å	*T* = 296 K
β = 120.585 (2)°	Block, colourless
*V* = 3532.35 (16) Å^3^	0.21 × 0.16 × 0.09 mm
*Z* = 2	

### Data collection

**Table:**

Bruker APEXII CCD diffractometer	6213 independent reflections
Radiation source: fine-focus sealed tube	4431 reflections with *I* > 2σ(*I*)
graphite	*R*_int_ = 0.051
φ and ω scans	θ_max_ = 25.0°, θ_min_ = 1.9°
Absorption correction: multi-scan (*SADABS*; Sheldrick, 1996)	*h* = −13→12
*T*_min_ = 0.685, *T*_max_ = 0.834	*k* = −24→30
24708 measured reflections	*l* = −16→16

### Refinement

**Table:**

Refinement on *F*^2^	Primary atom site location: structure-invariant direct methods
Least-squares matrix: full	Secondary atom site location: difference Fourier map
*R*[*F*^2^ > 2σ(*F*^2^)] = 0.033	Hydrogen site location: inferred from neighbouring sites
*wR*(*F*^2^) = 0.073	H-atom parameters constrained
*S* = 1.01	*w* = 1/[σ^2^(*F*_o_^2^) + (0.0302*P*)^2^] where *P* = (*F*_o_^2^ + 2*F*_c_^2^)/3
6213 reflections	(Δ/σ)_max_ < 0.001
464 parameters	Δρ_max_ = 0.72 e Å^−^^3^
0 restraints	Δρ_min_ = −0.81 e Å^−^^3^

### Special details

**Table:**

Geometry. All e.s.d.'s (except the e.s.d. in the dihedral angle between two l.s. planes) are estimated using the full covariance matrix. The cell e.s.d.'s are taken into account individually in the estimation of e.s.d.'s in distances, angles and torsion angles; correlations between e.s.d.'s in cell parameters are only used when they are defined by crystal symmetry. An approximate (isotropic) treatment of cell e.s.d.'s is used for estimating e.s.d.'s involving l.s. planes.
Refinement. Refinement of *F*^2^ against ALL reflections. The weighted *R*-factor *wR* and goodness of fit *S* are based on *F*^2^, conventional *R*-factors *R* are based on *F*, with *F* set to zero for negative *F*^2^. The threshold expression of *F*^2^ > σ(*F*^2^) is used only for calculating *R*-factors(gt) *etc*. and is not relevant to the choice of reflections for refinement. *R*-factors based on *F*^2^ are statistically about twice as large as those based on *F*, and *R*- factors based on ALL data will be even larger.

### Fractional atomic coordinates and isotropic or equivalent isotropic displacement parameters (Å^2^)

**Table:**

	*x*	*y*	*z*	*U*_iso_*/*U*_eq_	
Tb1	0.046508 (18)	−0.002691 (7)	0.159782 (15)	0.02642 (8)	
O1	−0.0327 (3)	−0.08517 (10)	0.1979 (2)	0.0380 (7)	
O2	−0.1196 (3)	−0.01283 (11)	0.2169 (2)	0.0433 (8)	
O3	−0.0956 (3)	−0.13390 (12)	0.3442 (3)	0.0525 (9)	
O4	0.1664 (3)	−0.06735 (10)	0.1225 (2)	0.0345 (7)	
O6	0.3286 (3)	−0.15208 (12)	0.1539 (3)	0.0552 (9)	
O7	0.2552 (3)	0.04186 (10)	0.1987 (2)	0.0356 (7)	
O8	0.1174 (3)	0.03129 (10)	0.0185 (2)	0.0328 (7)	
O9	0.4080 (3)	0.11053 (10)	0.1575 (2)	0.0420 (7)	
N1	0.1196 (3)	0.06788 (12)	0.3146 (3)	0.0328 (8)	
N2	0.2183 (3)	−0.03039 (13)	0.3569 (3)	0.0324 (8)	
C1	−0.1022 (4)	−0.06115 (17)	0.2289 (3)	0.0362 (10)	
C2	−0.1734 (4)	−0.09094 (17)	0.2798 (4)	0.0427 (11)	
H2	−0.1920	−0.0677	0.3263	0.051*	
C3	−0.3045 (5)	−0.1141 (2)	0.1872 (4)	0.0663 (16)	
H3A	−0.3503	−0.1315	0.2196	0.099*	
H3B	−0.2848	−0.1383	0.1448	0.099*	
H3C	−0.3612	−0.0869	0.1385	0.099*	
C4	0.0131 (5)	−0.12456 (18)	0.4496 (4)	0.0439 (12)	
C5	0.0494 (5)	−0.0768 (2)	0.5007 (4)	0.0534 (13)	
H5	0.0031	−0.0471	0.4625	0.064*	
C6	0.1566 (5)	−0.0739 (2)	0.6107 (4)	0.0627 (15)	
H6	0.1809	−0.0419	0.6464	0.075*	
C7	0.2266 (6)	−0.1169 (3)	0.6671 (4)	0.0693 (17)	
H7	0.2978	−0.1143	0.7408	0.083*	
C8	0.1909 (6)	−0.1642 (2)	0.6140 (5)	0.0674 (16)	
H8	0.2395	−0.1936	0.6517	0.081*	
C9	0.0842 (5)	−0.16841 (19)	0.5056 (4)	0.0563 (14)	
H9	0.0603	−0.2005	0.4703	0.068*	
C10	0.1568 (4)	−0.08626 (15)	0.0355 (4)	0.0331 (10)	
C11	0.2390 (4)	−0.13420 (16)	0.0440 (4)	0.0403 (11)	
H11	0.1756	−0.1622	0.0022	0.048*	
C12	0.3243 (5)	−0.12405 (19)	−0.0090 (4)	0.0591 (14)	
H12A	0.3722	−0.1551	−0.0062	0.089*	
H12B	0.3881	−0.0969	0.0313	0.089*	
H12C	0.2667	−0.1138	−0.0857	0.089*	
C13	0.2775 (6)	−0.18248 (16)	0.2065 (4)	0.0499 (13)	
C14	0.3778 (6)	−0.20956 (19)	0.2975 (5)	0.0693 (16)	
H14	0.4675	−0.2080	0.3152	0.083*	
C15	0.3408 (9)	−0.2388 (2)	0.3610 (5)	0.095 (2)	
H15	0.4063	−0.2574	0.4223	0.114*	
C16	0.2108 (10)	−0.2408 (3)	0.3352 (7)	0.106 (3)	
H16	0.1873	−0.2607	0.3786	0.127*	
C17	0.1134 (7)	−0.2138 (2)	0.2457 (6)	0.0821 (19)	
H17	0.0240	−0.2153	0.2288	0.099*	
C18	0.1462 (6)	−0.18462 (18)	0.1811 (4)	0.0566 (14)	
H18	0.0795	−0.1663	0.1201	0.068*	
C19	0.2271 (4)	0.04761 (14)	0.1005 (4)	0.0290 (9)	
C20	0.3272 (4)	0.07338 (16)	0.0744 (3)	0.0350 (10)	
H20	0.2781	0.0902	0.0008	0.042*	
C21	0.4238 (5)	0.03316 (18)	0.0743 (4)	0.0519 (13)	
H21A	0.4882	0.0498	0.0600	0.078*	
H21B	0.3735	0.0079	0.0169	0.078*	
H21C	0.4705	0.0163	0.1460	0.078*	
C22	0.3444 (5)	0.15387 (16)	0.1655 (4)	0.0421 (11)	
C23	0.2139 (6)	0.16749 (19)	0.0909 (5)	0.0788 (19)	
H23	0.1596	0.1463	0.0299	0.095*	
C24	0.1635 (7)	0.2129 (2)	0.1072 (6)	0.105 (2)	
H24	0.0756	0.2228	0.0545	0.126*	
C25	0.2377 (7)	0.2438 (2)	0.1973 (6)	0.093 (2)	
H25	0.2009	0.2739	0.2075	0.112*	
C26	0.3668 (7)	0.2296 (2)	0.2725 (5)	0.0803 (18)	
H26	0.4192	0.2503	0.3350	0.096*	
C27	0.4214 (5)	0.18466 (19)	0.2569 (4)	0.0612 (14)	
H27	0.5102	0.1754	0.3085	0.073*	
C28	0.0718 (5)	0.11558 (16)	0.2958 (4)	0.0424 (11)	
H28	−0.0033	0.1230	0.2260	0.051*	
C29	0.1269 (5)	0.15552 (17)	0.3740 (4)	0.0534 (13)	
H29	0.0896	0.1886	0.3566	0.064*	
C30	0.2360 (5)	0.14519 (18)	0.4762 (4)	0.0502 (13)	
H30	0.2738	0.1713	0.5298	0.060*	
C31	0.4063 (5)	0.08146 (19)	0.6069 (4)	0.0457 (12)	
H31	0.4490	0.1067	0.6619	0.055*	
C32	0.4537 (5)	0.03306 (19)	0.6286 (4)	0.0442 (12)	
H32	0.5291	0.0254	0.6980	0.053*	
C33	0.4339 (4)	−0.05845 (18)	0.5669 (4)	0.0424 (11)	
H33	0.5067	−0.0682	0.6360	0.051*	
C34	0.3696 (4)	−0.09424 (18)	0.4848 (4)	0.0434 (12)	
H34	0.3963	−0.1288	0.4978	0.052*	
C35	0.2631 (4)	−0.07870 (16)	0.3806 (3)	0.0367 (11)	
H35	0.2211	−0.1036	0.3247	0.044*	
C36	0.2919 (4)	0.09550 (17)	0.5014 (4)	0.0378 (11)	
C37	0.2292 (4)	0.05749 (15)	0.4173 (3)	0.0311 (10)	
C38	0.2803 (4)	0.00542 (15)	0.4403 (3)	0.0303 (9)	
C39	0.3908 (4)	−0.00710 (17)	0.5475 (4)	0.0366 (10)	
O5	0.0824 (3)	−0.07015 (10)	−0.0636 (2)	0.0376 (7)	

### Atomic displacement parameters (Å^2^)

**Table:**

	*U*^11^	*U*^22^	*U*^33^	*U*^12^	*U*^13^	*U*^23^
Tb1	0.02532 (12)	0.02833 (12)	0.02071 (12)	0.00011 (9)	0.00815 (9)	0.00056 (10)
O1	0.0371 (19)	0.0346 (17)	0.0387 (19)	−0.0005 (14)	0.0166 (16)	0.0042 (14)
O2	0.051 (2)	0.0367 (19)	0.049 (2)	−0.0009 (14)	0.0305 (18)	0.0037 (14)
O3	0.055 (2)	0.046 (2)	0.040 (2)	−0.0106 (17)	0.0126 (19)	0.0089 (16)
O4	0.0406 (19)	0.0335 (16)	0.0256 (17)	0.0054 (13)	0.0142 (15)	0.0005 (13)
O6	0.049 (2)	0.050 (2)	0.054 (2)	0.0101 (16)	0.017 (2)	0.0100 (17)
O7	0.0324 (18)	0.0422 (18)	0.0273 (17)	−0.0068 (13)	0.0116 (15)	−0.0016 (14)
O8	0.0284 (17)	0.0365 (17)	0.0211 (16)	−0.0065 (13)	0.0036 (15)	−0.0003 (13)
O9	0.0315 (18)	0.0368 (18)	0.048 (2)	−0.0069 (14)	0.0129 (16)	−0.0010 (15)
N1	0.032 (2)	0.034 (2)	0.030 (2)	0.0013 (16)	0.0137 (19)	−0.0011 (17)
N2	0.034 (2)	0.037 (2)	0.022 (2)	0.0028 (17)	0.0103 (18)	0.0033 (17)
C1	0.030 (3)	0.044 (3)	0.023 (3)	−0.004 (2)	0.006 (2)	0.004 (2)
C2	0.040 (3)	0.048 (3)	0.037 (3)	−0.005 (2)	0.017 (3)	0.010 (2)
C3	0.043 (3)	0.090 (4)	0.050 (3)	−0.026 (3)	0.012 (3)	0.017 (3)
C4	0.042 (3)	0.054 (3)	0.036 (3)	−0.003 (2)	0.020 (3)	0.012 (3)
C5	0.058 (3)	0.056 (3)	0.046 (3)	0.001 (3)	0.026 (3)	−0.002 (3)
C6	0.062 (4)	0.073 (4)	0.046 (4)	−0.011 (3)	0.023 (3)	−0.020 (3)
C7	0.051 (4)	0.114 (5)	0.041 (4)	0.000 (4)	0.022 (3)	0.013 (4)
C8	0.053 (4)	0.080 (4)	0.062 (4)	0.014 (3)	0.024 (3)	0.035 (3)
C9	0.057 (4)	0.051 (3)	0.051 (4)	−0.004 (3)	0.021 (3)	0.012 (3)
C10	0.028 (3)	0.031 (2)	0.033 (3)	0.0001 (19)	0.010 (2)	0.002 (2)
C11	0.040 (3)	0.037 (3)	0.032 (3)	0.013 (2)	0.009 (2)	0.001 (2)
C12	0.048 (3)	0.067 (4)	0.063 (4)	0.019 (3)	0.030 (3)	0.005 (3)
C13	0.069 (4)	0.027 (3)	0.052 (3)	0.008 (2)	0.031 (3)	0.006 (2)
C14	0.073 (4)	0.041 (3)	0.066 (4)	0.005 (3)	0.015 (4)	0.007 (3)
C15	0.132 (7)	0.062 (4)	0.048 (4)	0.001 (5)	0.015 (5)	0.021 (3)
C16	0.188 (9)	0.059 (4)	0.099 (6)	−0.001 (6)	0.093 (7)	0.011 (4)
C17	0.110 (6)	0.048 (4)	0.116 (6)	−0.004 (4)	0.078 (5)	−0.003 (4)
C18	0.069 (4)	0.043 (3)	0.058 (4)	0.004 (3)	0.032 (3)	0.003 (3)
C19	0.027 (3)	0.024 (2)	0.032 (3)	0.0024 (18)	0.012 (2)	−0.003 (2)
C20	0.030 (3)	0.039 (3)	0.030 (3)	−0.002 (2)	0.011 (2)	−0.001 (2)
C21	0.046 (3)	0.061 (3)	0.053 (3)	0.000 (2)	0.029 (3)	−0.002 (3)
C22	0.036 (3)	0.028 (2)	0.054 (3)	0.000 (2)	0.017 (3)	−0.001 (2)
C23	0.053 (4)	0.040 (3)	0.093 (5)	0.011 (3)	0.001 (4)	−0.012 (3)
C24	0.074 (5)	0.057 (4)	0.124 (6)	0.017 (3)	0.007 (4)	−0.019 (4)
C25	0.092 (5)	0.046 (4)	0.111 (6)	0.015 (4)	0.029 (5)	−0.020 (4)
C26	0.084 (5)	0.059 (4)	0.069 (4)	−0.012 (3)	0.018 (4)	−0.023 (3)
C27	0.055 (4)	0.053 (3)	0.055 (4)	−0.002 (3)	0.014 (3)	−0.002 (3)
C28	0.046 (3)	0.040 (3)	0.031 (3)	0.005 (2)	0.013 (2)	−0.003 (2)
C29	0.065 (4)	0.037 (3)	0.052 (3)	0.005 (2)	0.025 (3)	−0.008 (3)
C30	0.055 (3)	0.046 (3)	0.042 (3)	−0.011 (3)	0.020 (3)	−0.017 (3)
C31	0.043 (3)	0.065 (3)	0.024 (3)	−0.019 (3)	0.013 (2)	−0.019 (2)
C32	0.041 (3)	0.056 (3)	0.024 (3)	−0.006 (2)	0.008 (2)	−0.002 (2)
C33	0.037 (3)	0.055 (3)	0.023 (3)	0.001 (2)	0.007 (2)	0.009 (2)
C34	0.046 (3)	0.046 (3)	0.033 (3)	0.016 (2)	0.016 (3)	0.013 (2)
C35	0.042 (3)	0.037 (3)	0.025 (3)	−0.001 (2)	0.013 (2)	−0.001 (2)
C36	0.038 (3)	0.045 (3)	0.028 (3)	−0.011 (2)	0.015 (2)	−0.010 (2)
C37	0.030 (3)	0.038 (2)	0.027 (3)	−0.005 (2)	0.015 (2)	−0.002 (2)
C38	0.029 (2)	0.040 (2)	0.025 (2)	−0.002 (2)	0.016 (2)	0.004 (2)
C39	0.029 (3)	0.052 (3)	0.027 (2)	−0.002 (2)	0.012 (2)	0.005 (2)
O5	0.0440 (19)	0.0365 (17)	0.0244 (17)	0.0084 (14)	0.0116 (15)	0.0010 (14)

### Geometric parameters (Å, °)

**Table:**

Tb1—O8^i^	2.336 (3)	C12—H12C	0.9600
Tb1—O5^i^	2.344 (3)	C13—C18	1.363 (6)
Tb1—O4	2.381 (3)	C13—C14	1.388 (7)
Tb1—O2	2.422 (3)	C14—C15	1.381 (8)
Tb1—O7	2.457 (3)	C14—H14	0.9300
Tb1—O1	2.475 (3)	C15—C16	1.346 (9)
Tb1—N2	2.525 (3)	C15—H15	0.9300
Tb1—N1	2.606 (3)	C16—C17	1.364 (8)
Tb1—O8	2.623 (2)	C16—H16	0.9300
Tb1—C1	2.787 (4)	C17—C18	1.362 (7)
Tb1—C19	2.896 (4)	C17—H17	0.9300
Tb1—Tb1^i^	3.9935 (4)	C18—H18	0.9300
O1—C1	1.246 (5)	C19—C20	1.521 (5)
O2—C1	1.261 (5)	C20—C21	1.519 (5)
O3—C4	1.375 (5)	C20—H20	0.9800
O3—C2	1.418 (5)	C21—H21A	0.9600
O4—C10	1.252 (4)	C21—H21B	0.9600
O6—C13	1.387 (5)	C21—H21C	0.9600
O6—C11	1.412 (5)	C22—C23	1.363 (6)
O7—C19	1.237 (4)	C22—C27	1.371 (6)
O8—C19	1.264 (4)	C23—C24	1.376 (7)
O8—Tb1^i^	2.336 (3)	C23—H23	0.9300
O9—C22	1.370 (5)	C24—C25	1.355 (7)
O9—C20	1.421 (4)	C24—H24	0.9300
N1—C28	1.319 (5)	C25—C26	1.358 (7)
N1—C37	1.363 (5)	C25—H25	0.9300
N2—C35	1.325 (5)	C26—C27	1.387 (7)
N2—C38	1.363 (5)	C26—H26	0.9300
C1—C2	1.530 (5)	C27—H27	0.9300
C2—C3	1.517 (6)	C28—C29	1.393 (6)
C2—H2	0.9800	C28—H28	0.9300
C3—H3A	0.9600	C29—C30	1.356 (6)
C3—H3B	0.9600	C29—H29	0.9300
C3—H3C	0.9600	C30—C36	1.397 (6)
C4—C5	1.377 (6)	C30—H30	0.9300
C4—C9	1.380 (6)	C31—C32	1.334 (6)
C5—C6	1.390 (6)	C31—C36	1.428 (6)
C5—H5	0.9300	C31—H31	0.9300
C6—C7	1.359 (7)	C32—C39	1.426 (6)
C6—H6	0.9300	C32—H32	0.9300
C7—C8	1.377 (7)	C33—C34	1.355 (6)
C7—H7	0.9300	C33—C39	1.392 (6)
C8—C9	1.377 (6)	C33—H33	0.9300
C8—H8	0.9300	C34—C35	1.394 (5)
C9—H9	0.9300	C34—H34	0.9300
C10—O5	1.262 (4)	C35—H35	0.9300
C10—C11	1.524 (5)	C36—C37	1.409 (5)
C11—C12	1.516 (6)	C37—C38	1.436 (5)
C11—H11	0.9800	C38—C39	1.414 (6)
C12—H12A	0.9600	O5—Tb1^i^	2.344 (3)
C12—H12B	0.9600		
			
O8^i^—Tb1—O5^i^	73.53 (9)	C9—C8—C7	120.7 (5)
O8^i^—Tb1—O4	78.03 (9)	C9—C8—H8	119.6
O5^i^—Tb1—O4	134.94 (9)	C7—C8—H8	119.6
O8^i^—Tb1—O2	88.12 (9)	C8—C9—C4	119.5 (5)
O5^i^—Tb1—O2	84.17 (9)	C8—C9—H9	120.3
O4—Tb1—O2	129.28 (9)	C4—C9—H9	120.3
O8^i^—Tb1—O7	123.42 (9)	O4—C10—O5	126.8 (4)
O5^i^—Tb1—O7	90.70 (9)	O4—C10—C11	119.6 (4)
O4—Tb1—O7	76.66 (9)	O5—C10—C11	113.6 (4)
O2—Tb1—O7	145.12 (9)	O6—C11—C12	106.5 (4)
O8^i^—Tb1—O1	76.59 (9)	O6—C11—C10	115.5 (3)
O5^i^—Tb1—O1	128.11 (9)	C12—C11—C10	110.5 (4)
O4—Tb1—O1	76.04 (9)	O6—C11—H11	108.1
O2—Tb1—O1	53.26 (9)	C12—C11—H11	108.1
O7—Tb1—O1	141.13 (9)	C10—C11—H11	108.1
O8^i^—Tb1—N2	145.04 (10)	C11—C12—H12A	109.5
O5^i^—Tb1—N2	139.61 (10)	C11—C12—H12B	109.5
O4—Tb1—N2	79.43 (9)	H12A—C12—H12B	109.5
O2—Tb1—N2	85.83 (10)	C11—C12—H12C	109.5
O7—Tb1—N2	75.96 (10)	H12A—C12—H12C	109.5
O1—Tb1—N2	72.25 (10)	H12B—C12—H12C	109.5
O8^i^—Tb1—N1	147.14 (10)	C18—C13—O6	126.5 (5)
O5^i^—Tb1—N1	75.70 (10)	C18—C13—C14	120.7 (5)
O4—Tb1—N1	133.55 (10)	O6—C13—C14	112.7 (5)
O2—Tb1—N1	77.51 (10)	C15—C14—C13	118.2 (6)
O7—Tb1—N1	67.82 (9)	C15—C14—H14	120.9
O1—Tb1—N1	115.16 (9)	C13—C14—H14	120.9
N2—Tb1—N1	63.94 (10)	C16—C15—C14	120.6 (7)
O8^i^—Tb1—O8	72.87 (10)	C16—C15—H15	119.7
O5^i^—Tb1—O8	69.72 (9)	C14—C15—H15	119.7
O4—Tb1—O8	68.89 (8)	C15—C16—C17	120.5 (7)
O2—Tb1—O8	151.00 (10)	C15—C16—H16	119.7
O7—Tb1—O8	50.91 (8)	C17—C16—H16	119.7
O1—Tb1—O8	137.20 (9)	C18—C17—C16	120.4 (6)
N2—Tb1—O8	122.24 (9)	C18—C17—H17	119.8
N1—Tb1—O8	106.74 (9)	C16—C17—H17	119.8
O8^i^—Tb1—C1	83.55 (10)	C17—C18—C13	119.5 (5)
O5^i^—Tb1—C1	108.06 (11)	C17—C18—H18	120.3
O4—Tb1—C1	102.55 (11)	C13—C18—H18	120.3
O2—Tb1—C1	26.86 (10)	O7—C19—O8	122.1 (4)
O7—Tb1—C1	151.27 (10)	O7—C19—C20	120.5 (4)
O1—Tb1—C1	26.56 (10)	O8—C19—C20	117.3 (4)
N2—Tb1—C1	75.69 (11)	O7—C19—Tb1	57.2 (2)
N1—Tb1—C1	95.31 (11)	O8—C19—Tb1	64.9 (2)
O8—Tb1—C1	156.04 (10)	C20—C19—Tb1	177.5 (3)
O8^i^—Tb1—C19	98.59 (11)	O9—C20—C21	106.7 (3)
O5^i^—Tb1—C19	79.34 (10)	O9—C20—C19	111.2 (3)
O4—Tb1—C19	71.15 (9)	C21—C20—C19	109.9 (3)
O2—Tb1—C19	159.56 (10)	O9—C20—H20	109.7
O7—Tb1—C19	25.05 (9)	C21—C20—H20	109.7
O1—Tb1—C19	147.09 (10)	C19—C20—H20	109.7
N2—Tb1—C19	98.96 (11)	C20—C21—H21A	109.5
N1—Tb1—C19	86.73 (10)	C20—C21—H21B	109.5
O8—Tb1—C19	25.87 (9)	H21A—C21—H21B	109.5
C1—Tb1—C19	172.60 (12)	C20—C21—H21C	109.5
O8^i^—Tb1—Tb1^i^	38.89 (6)	H21A—C21—H21C	109.5
O5^i^—Tb1—Tb1^i^	66.82 (6)	H21B—C21—H21C	109.5
O4—Tb1—Tb1^i^	69.02 (6)	C23—C22—O9	125.1 (4)
O2—Tb1—Tb1^i^	123.73 (7)	C23—C22—C27	119.6 (5)
O7—Tb1—Tb1^i^	84.72 (6)	O9—C22—C27	115.4 (4)
O1—Tb1—Tb1^i^	110.26 (6)	C22—C23—C24	119.1 (5)
N2—Tb1—Tb1^i^	146.08 (7)	C22—C23—H23	120.4
N1—Tb1—Tb1^i^	132.89 (7)	C24—C23—H23	120.4
O8—Tb1—Tb1^i^	33.99 (6)	C25—C24—C23	122.2 (6)
C1—Tb1—Tb1^i^	122.32 (8)	C25—C24—H24	118.9
C19—Tb1—Tb1^i^	59.75 (9)	C23—C24—H24	118.9
C1—O1—Tb1	90.8 (2)	C24—C25—C26	118.5 (6)
C1—O2—Tb1	92.9 (2)	C24—C25—H25	120.7
C4—O3—C2	118.1 (3)	C26—C25—H25	120.7
C10—O4—Tb1	134.8 (3)	C25—C26—C27	120.6 (5)
C13—O6—C11	118.8 (4)	C25—C26—H26	119.7
C19—O7—Tb1	97.8 (2)	C27—C26—H26	119.7
C19—O8—Tb1^i^	162.5 (3)	C22—C27—C26	119.9 (5)
C19—O8—Tb1	89.2 (2)	C22—C27—H27	120.0
Tb1^i^—O8—Tb1	107.13 (10)	C26—C27—H27	120.0
C22—O9—C20	117.5 (3)	N1—C28—C29	124.0 (4)
C28—N1—C37	117.4 (4)	N1—C28—H28	118.0
C28—N1—Tb1	124.2 (3)	C29—C28—H28	118.0
C37—N1—Tb1	117.5 (2)	C30—C29—C28	118.6 (4)
C35—N2—C38	117.3 (4)	C30—C29—H29	120.7
C35—N2—Tb1	121.7 (3)	C28—C29—H29	120.7
C38—N2—Tb1	120.5 (3)	C29—C30—C36	120.3 (4)
O1—C1—O2	122.2 (4)	C29—C30—H30	119.9
O1—C1—C2	119.6 (4)	C36—C30—H30	119.9
O2—C1—C2	118.1 (4)	C32—C31—C36	121.7 (4)
O1—C1—Tb1	62.6 (2)	C32—C31—H31	119.2
O2—C1—Tb1	60.2 (2)	C36—C31—H31	119.2
C2—C1—Tb1	173.7 (3)	C31—C32—C39	121.2 (4)
O3—C2—C3	105.1 (4)	C31—C32—H32	119.4
O3—C2—C1	111.8 (4)	C39—C32—H32	119.4
C3—C2—C1	109.9 (3)	C34—C33—C39	119.9 (4)
O3—C2—H2	110.0	C34—C33—H33	120.0
C3—C2—H2	110.0	C39—C33—H33	120.0
C1—C2—H2	110.0	C33—C34—C35	119.2 (4)
C2—C3—H3A	109.5	C33—C34—H34	120.4
C2—C3—H3B	109.5	C35—C34—H34	120.4
H3A—C3—H3B	109.5	N2—C35—C34	123.6 (4)
C2—C3—H3C	109.5	N2—C35—H35	118.2
H3A—C3—H3C	109.5	C34—C35—H35	118.2
H3B—C3—H3C	109.5	C30—C36—C37	117.2 (4)
O3—C4—C5	125.2 (4)	C30—C36—C31	123.7 (4)
O3—C4—C9	114.2 (4)	C37—C36—C31	119.1 (4)
C5—C4—C9	120.5 (5)	N1—C37—C36	122.6 (4)
C4—C5—C6	118.7 (5)	N1—C37—C38	118.1 (4)
C4—C5—H5	120.7	C36—C37—C38	119.3 (4)
C6—C5—H5	120.7	N2—C38—C39	122.5 (4)
C7—C6—C5	121.4 (5)	N2—C38—C37	117.9 (4)
C7—C6—H6	119.3	C39—C38—C37	119.6 (4)
C5—C6—H6	119.3	C33—C39—C38	117.4 (4)
C6—C7—C8	119.3 (5)	C33—C39—C32	123.5 (4)
C6—C7—H7	120.4	C38—C39—C32	119.0 (4)
C8—C7—H7	120.4	C10—O5—Tb1^i^	139.8 (2)
			
O8^i^—Tb1—O1—C1	−102.6 (2)	O7—Tb1—C1—O2	100.1 (3)
O5^i^—Tb1—O1—C1	−46.5 (3)	O1—Tb1—C1—O2	−171.4 (4)
O4—Tb1—O1—C1	176.6 (2)	N2—Tb1—C1—O2	109.7 (3)
O2—Tb1—O1—C1	−4.8 (2)	N1—Tb1—C1—O2	48.3 (3)
O7—Tb1—O1—C1	130.0 (2)	O8—Tb1—C1—O2	−108.9 (3)
N2—Tb1—O1—C1	93.4 (2)	Tb1^i^—Tb1—C1—O2	−101.8 (2)
N1—Tb1—O1—C1	44.7 (3)	C4—O3—C2—C3	−163.7 (4)
O8—Tb1—O1—C1	−148.0 (2)	C4—O3—C2—C1	77.1 (5)
C19—Tb1—O1—C1	172.3 (2)	O1—C1—C2—O3	34.8 (5)
Tb1^i^—Tb1—O1—C1	−122.4 (2)	O2—C1—C2—O3	−147.2 (4)
O8^i^—Tb1—O2—C1	79.4 (2)	O1—C1—C2—C3	−81.4 (5)
O5^i^—Tb1—O2—C1	153.0 (3)	O2—C1—C2—C3	96.6 (5)
O4—Tb1—O2—C1	6.6 (3)	C2—O3—C4—C5	3.5 (6)
O7—Tb1—O2—C1	−124.1 (2)	C2—O3—C4—C9	−179.0 (4)
O1—Tb1—O2—C1	4.8 (2)	O3—C4—C5—C6	175.6 (4)
N2—Tb1—O2—C1	−66.1 (2)	C9—C4—C5—C6	−1.7 (7)
N1—Tb1—O2—C1	−130.4 (3)	C4—C5—C6—C7	1.0 (7)
O8—Tb1—O2—C1	127.6 (3)	C5—C6—C7—C8	0.3 (8)
C19—Tb1—O2—C1	−170.7 (3)	C6—C7—C8—C9	−1.1 (8)
Tb1^i^—Tb1—O2—C1	96.0 (2)	C7—C8—C9—C4	0.5 (7)
O8^i^—Tb1—O4—C10	26.0 (3)	O3—C4—C9—C8	−176.6 (4)
O5^i^—Tb1—O4—C10	−25.6 (4)	C5—C4—C9—C8	0.9 (7)
O2—Tb1—O4—C10	103.5 (4)	Tb1—O4—C10—O5	9.0 (6)
O7—Tb1—O4—C10	−103.0 (4)	Tb1—O4—C10—C11	−170.0 (3)
O1—Tb1—O4—C10	105.0 (4)	C13—O6—C11—C12	−155.5 (4)
N2—Tb1—O4—C10	179.1 (4)	C13—O6—C11—C10	81.4 (5)
N1—Tb1—O4—C10	−143.5 (3)	O4—C10—C11—O6	−3.8 (6)
O8—Tb1—O4—C10	−50.1 (3)	O5—C10—C11—O6	177.1 (3)
C1—Tb1—O4—C10	106.5 (4)	O4—C10—C11—C12	−124.7 (4)
C19—Tb1—O4—C10	−77.5 (4)	O5—C10—C11—C12	56.2 (5)
Tb1^i^—Tb1—O4—C10	−13.6 (3)	C11—O6—C13—C18	−22.1 (7)
O8^i^—Tb1—O7—C19	8.2 (3)	C11—O6—C13—C14	162.3 (4)
O5^i^—Tb1—O7—C19	−62.4 (2)	C18—C13—C14—C15	0.3 (8)
O4—Tb1—O7—C19	73.9 (2)	O6—C13—C14—C15	176.2 (5)
O2—Tb1—O7—C19	−143.2 (2)	C13—C14—C15—C16	−0.2 (10)
O1—Tb1—O7—C19	120.3 (2)	C14—C15—C16—C17	−0.1 (11)
N2—Tb1—O7—C19	156.1 (2)	C15—C16—C17—C18	0.2 (10)
N1—Tb1—O7—C19	−136.6 (2)	C16—C17—C18—C13	−0.1 (8)
O8—Tb1—O7—C19	0.4 (2)	O6—C13—C18—C17	−175.4 (4)
C1—Tb1—O7—C19	165.8 (3)	C14—C13—C18—C17	−0.1 (7)
Tb1^i^—Tb1—O7—C19	4.3 (2)	Tb1—O7—C19—O8	−0.8 (4)
O8^i^—Tb1—O8—C19	−173.6 (3)	Tb1—O7—C19—C20	−178.8 (3)
O5^i^—Tb1—O8—C19	108.1 (2)	Tb1^i^—O8—C19—O7	−158.5 (6)
O4—Tb1—O8—C19	−90.1 (2)	Tb1—O8—C19—O7	0.8 (4)
O2—Tb1—O8—C19	135.2 (2)	Tb1^i^—O8—C19—C20	19.5 (10)
O7—Tb1—O8—C19	−0.4 (2)	Tb1—O8—C19—C20	178.8 (3)
O1—Tb1—O8—C19	−127.2 (2)	Tb1^i^—O8—C19—Tb1	−159.3 (9)
N2—Tb1—O8—C19	−28.6 (2)	O8^i^—Tb1—C19—O7	−173.1 (2)
N1—Tb1—O8—C19	40.8 (2)	O5^i^—Tb1—C19—O7	115.6 (2)
C1—Tb1—O8—C19	−163.0 (3)	O4—Tb1—C19—O7	−98.9 (2)
Tb1^i^—Tb1—O8—C19	−173.6 (3)	O2—Tb1—C19—O7	78.9 (4)
O8^i^—Tb1—O8—Tb1^i^	0.0	O1—Tb1—C19—O7	−94.5 (3)
O5^i^—Tb1—O8—Tb1^i^	−78.33 (11)	N2—Tb1—C19—O7	−23.4 (2)
O4—Tb1—O8—Tb1^i^	83.47 (11)	N1—Tb1—C19—O7	39.6 (2)
O2—Tb1—O8—Tb1^i^	−51.2 (2)	O8—Tb1—C19—O7	−179.2 (4)
O7—Tb1—O8—Tb1^i^	173.19 (16)	Tb1^i^—Tb1—C19—O7	−175.1 (2)
O1—Tb1—O8—Tb1^i^	46.38 (17)	O8^i^—Tb1—C19—O8	6.2 (3)
N2—Tb1—O8—Tb1^i^	145.04 (11)	O5^i^—Tb1—C19—O8	−65.1 (2)
N1—Tb1—O8—Tb1^i^	−145.60 (11)	O4—Tb1—C19—O8	80.3 (2)
C1—Tb1—O8—Tb1^i^	10.6 (3)	O2—Tb1—C19—O8	−101.9 (4)
C19—Tb1—O8—Tb1^i^	173.6 (3)	O7—Tb1—C19—O8	179.2 (4)
O8^i^—Tb1—N1—C28	−21.7 (4)	O1—Tb1—C19—O8	84.7 (3)
O5^i^—Tb1—N1—C28	−0.8 (3)	N2—Tb1—C19—O8	155.8 (2)
O4—Tb1—N1—C28	139.0 (3)	N1—Tb1—C19—O8	−141.2 (2)
O2—Tb1—N1—C28	−87.9 (3)	Tb1^i^—Tb1—C19—O8	4.13 (17)
O7—Tb1—N1—C28	96.0 (3)	C22—O9—C20—C21	173.4 (3)
O1—Tb1—N1—C28	−126.5 (3)	C22—O9—C20—C19	−66.8 (4)
N2—Tb1—N1—C28	−179.3 (3)	O7—C19—C20—O9	−30.5 (5)
O8—Tb1—N1—C28	62.5 (3)	O8—C19—C20—O9	151.5 (3)
C1—Tb1—N1—C28	−108.1 (3)	O7—C19—C20—C21	87.4 (4)
C19—Tb1—N1—C28	79.0 (3)	O8—C19—C20—C21	−90.6 (4)
Tb1^i^—Tb1—N1—C28	36.9 (4)	C20—O9—C22—C23	−9.0 (7)
O8^i^—Tb1—N1—C37	169.4 (2)	C20—O9—C22—C27	170.6 (4)
O5^i^—Tb1—N1—C37	−169.6 (3)	O9—C22—C23—C24	−178.2 (5)
O4—Tb1—N1—C37	−29.9 (3)	C27—C22—C23—C24	2.2 (9)
O2—Tb1—N1—C37	103.3 (3)	C22—C23—C24—C25	−2.6 (11)
O7—Tb1—N1—C37	−72.9 (3)	C23—C24—C25—C26	1.4 (11)
O1—Tb1—N1—C37	64.6 (3)	C24—C25—C26—C27	0.2 (10)
N2—Tb1—N1—C37	11.8 (2)	C23—C22—C27—C26	−0.7 (8)
O8—Tb1—N1—C37	−106.4 (3)	O9—C22—C27—C26	179.7 (5)
C1—Tb1—N1—C37	83.0 (3)	C25—C26—C27—C22	−0.5 (9)
C19—Tb1—N1—C37	−89.8 (3)	C37—N1—C28—C29	0.3 (6)
Tb1^i^—Tb1—N1—C37	−131.9 (2)	Tb1—N1—C28—C29	−168.6 (3)
O8^i^—Tb1—N2—C35	17.8 (4)	N1—C28—C29—C30	−0.2 (7)
O5^i^—Tb1—N2—C35	174.5 (3)	C28—C29—C30—C36	0.5 (7)
O4—Tb1—N2—C35	−32.7 (3)	C36—C31—C32—C39	−0.7 (7)
O2—Tb1—N2—C35	98.5 (3)	C39—C33—C34—C35	1.7 (6)
O7—Tb1—N2—C35	−111.5 (3)	C38—N2—C35—C34	−0.6 (6)
O1—Tb1—N2—C35	45.8 (3)	Tb1—N2—C35—C34	171.7 (3)
N1—Tb1—N2—C35	176.6 (3)	C33—C34—C35—N2	−1.2 (6)
O8—Tb1—N2—C35	−89.3 (3)	C29—C30—C36—C37	−0.7 (6)
C1—Tb1—N2—C35	73.3 (3)	C29—C30—C36—C31	−179.9 (4)
C19—Tb1—N2—C35	−101.5 (3)	C32—C31—C36—C30	177.8 (4)
Tb1^i^—Tb1—N2—C35	−54.3 (4)	C32—C31—C36—C37	−1.4 (6)
O8^i^—Tb1—N2—C38	−170.1 (2)	C28—N1—C37—C36	−0.6 (5)
O5^i^—Tb1—N2—C38	−13.5 (3)	Tb1—N1—C37—C36	169.1 (3)
O4—Tb1—N2—C38	139.3 (3)	C28—N1—C37—C38	178.4 (3)
O2—Tb1—N2—C38	−89.4 (3)	Tb1—N1—C37—C38	−12.0 (4)
O7—Tb1—N2—C38	60.6 (3)	C30—C36—C37—N1	0.8 (6)
O1—Tb1—N2—C38	−142.1 (3)	C31—C36—C37—N1	180.0 (4)
N1—Tb1—N2—C38	−11.3 (2)	C30—C36—C37—C38	−178.1 (4)
O8—Tb1—N2—C38	82.8 (3)	C31—C36—C37—C38	1.1 (6)
C1—Tb1—N2—C38	−114.7 (3)	C35—N2—C38—C39	1.9 (5)
C19—Tb1—N2—C38	70.6 (3)	Tb1—N2—C38—C39	−170.5 (3)
Tb1^i^—Tb1—N2—C38	117.8 (2)	C35—N2—C38—C37	−177.4 (3)
Tb1—O1—C1—O2	8.8 (4)	Tb1—N2—C38—C37	10.2 (4)
Tb1—O1—C1—C2	−173.3 (3)	N1—C37—C38—N2	1.6 (5)
Tb1—O2—C1—O1	−9.0 (4)	C36—C37—C38—N2	−179.4 (3)
Tb1—O2—C1—C2	173.1 (3)	N1—C37—C38—C39	−177.7 (3)
O8^i^—Tb1—C1—O1	72.8 (2)	C36—C37—C38—C39	1.3 (5)
O5^i^—Tb1—C1—O1	143.1 (2)	C34—C33—C39—C38	−0.5 (6)
O4—Tb1—C1—O1	−3.4 (2)	C34—C33—C39—C32	−179.2 (4)
O2—Tb1—C1—O1	171.4 (4)	N2—C38—C39—C33	−1.3 (6)
O7—Tb1—C1—O1	−88.5 (3)	C37—C38—C39—C33	177.9 (3)
N2—Tb1—C1—O1	−78.9 (2)	N2—C38—C39—C32	177.4 (3)
N1—Tb1—C1—O1	−140.2 (2)	C37—C38—C39—C32	−3.3 (5)
O8—Tb1—C1—O1	62.6 (4)	C31—C32—C39—C33	−178.3 (4)
Tb1^i^—Tb1—C1—O1	69.6 (2)	C31—C32—C39—C38	3.1 (6)
O8^i^—Tb1—C1—O2	−98.6 (2)	O4—C10—O5—Tb1^i^	13.8 (7)
O5^i^—Tb1—C1—O2	−28.3 (3)	C11—C10—O5—Tb1^i^	−167.1 (3)
O4—Tb1—C1—O2	−174.8 (2)		

Symmetry codes: (i) −*x*, −*y*, −*z*.

## References

[bb1] Bruker (2006). *APEX2* and *SAINT* Bruker AXS Inc., Madison, Wisconsin, USA.

[bb2] Markus, D. M. & Buser, H. R. (1997). *Environ. Sci. Technol.* **31**, 1953–1959.

[bb3] Sheldrick, G. M. (1996). *SADABS* University of Göttingen, Germany.

[bb4] Sheldrick, G. M. (2008). *Acta Cryst.* A**64**, 112–122. 10.1107/S0108767307043930 18156677

[bb5] Zhao, N., Wang, S.-P., Ma, R.-X., Gao, Z.-H., Wang, R.-F. & Zhang, J.-J. (2008). *J. Alloys Compd*, **463**, 338–342.

